# Novel Approaches in the Inhibition of IgE-Induced Mast Cell Reactivity in Food Allergy

**DOI:** 10.3389/fimmu.2021.613461

**Published:** 2021-08-12

**Authors:** Chiara Tontini, Silvia Bulfone-Paus

**Affiliations:** Lydia Becker Institute for Immunology and Inflammation, Faculty of Biology, Medicine and Health, University of Manchester, Manchester, United Kingdom

**Keywords:** mast cells, IgE, desensitization, food allergy, biologics, immunotherapy, cytokines, inhibitory receptors

## Abstract

Allergy is an IgE-dependent type-I hypersensitivity reaction that can lead to life-threatening systemic symptoms such as anaphylaxis. In the pathogenesis of the allergic response, the common upstream event is the binding of allergens to specific IgE, inducing cross-linking of the high-affinity FcεRI on mast cells, triggering cellular degranulation and the release of histamine, proteases, lipids mediators, cytokines and chemokines with inflammatory activity. A number of novel therapeutic options to curb mast cell activation are in the pipeline for the treatment of severe allergies. In addition to anti-IgE therapy and allergen-specific immunotherapy, monoclonal antibodies targeted against several key Th2/alarmin cytokines (i.e. IL-4Rα, IL-33, TSLP), active modification of allergen-specific IgE (i.e. inhibitory compounds, monoclonal antibodies, de-sialylation), engagement of inhibitory receptors on mast cells and allergen-specific adjuvant vaccines, are new promising options to inhibit the uncontrolled release of mast cell mediators upon allergen exposure. In this review, we critically discuss the novel approaches targeting mast cells limiting allergic responses and the immunological mechanisms involved, with special interest on food allergy treatment.

## Introduction

Nowadays, over 20% of the world population actively suffers from one or more allergies, among which approximately 10% is living with food allergy (1, 2). Food allergies carry a high risk of developing systemic reactions upon allergen exposure, with 0.4–39.9% of allergic subjects experiencing at least one severe episode in their lifetime (3).

Anaphylaxis is a systemic reaction involving two or more organ systems, occurring shortly after the exposure to the culprit allergen. It manifests with a plethora of symptoms, including hives, angioedema, shortness of breath, vomiting, hypotension and cardiovascular collapse, which is potentially life-threatening and requires emergency treatment (4). The complex allergic reaction starts with the cross-linking of high-affinity immunoglobulin E (IgE) receptors (FcϵRI) expressed on effector cells such as mast cells (MCs) and basophils by IgE–allergen complexes. FcϵRI engagement causes cell degranulation and release of preformed mediators, such as amines (histamine, polyamines), proteoglycans (heparin, chondroitin sulphates, serglycin), proteases (tryptase, chymase-1, cathepsin G, granzyme B, carboxypeptidase A3), lysosomal enzymes (β-glucuronidase, β-hexosaminidase, arylsulfatase), newly formed lipid mediators (leukotrienes B4-C4, prostaglandin D2-E2), cytokines and chemokines (GM-CSF, IL-1β, IL-8, IL-13, MCP-1) (5, 6).

MC activation is also the cause of the delayed release of newly synthesized cytokines and chemokines (5, 6), that promote dendritic cell recruitment and activation (7, 8), T helper 2 (Th2) skewing (9–11), affinity maturation and epitope spreading on B and T cells (12, 13), additional IgE synthesis (14), and altogether the amplification of allergic responses (15). The release of vasoactive products, such as histamine, cysteinyl leukotrienes and platelet activating factor (16), serves as the main pathogenetic mechanism of anaphylaxis, which can lead to generalized cardiovascular involvement and collapse, the latter burdened with high mortality and morbidity (4).

In addition to their prominent role in the genesis of allergic and anaphylaxis symptoms, MCs actively participate to the complex interplay of innate and adaptive immunity in the defense against pathogens, wound healing and tumor surveillance (17–19). Due to the conspicuous array of surface receptors expressed, capable of sensing the surrounding environment and participate to immune recognition, MCs act as both initiators and suppressors of local immune responses (17, 20, 21). MCs engage in a bidirectional cross-talk with various immune cells, such as dendritic cells (10, 22–24), T cells (25) including T regulatory (Treg) cells (26–28), eosinophils (29, 30), B cells (31) and other cell types (17). Being capable of secreting both pro- and anti-inflammatory cytokines, like TNFα (7) and IL-10 (32), and several chemokines (6), MCs also contribute to the prevention and resolution of food allergy (33). Along with MCs, the above cell populations are considered equally important targets in food allergy treatment, however outside the main scope of the review and discussed elsewhere (34–36).

Strategies to pre-emptively curb MC activation are currently being explored for therapeutic purposes. Allergen-specific immunotherapy, recently developed biologics, a combination of both, and the discovery of new druggable targets are the most promising options available to treat food allergy.

The purpose of this review is to highlight the different immunological mechanisms targeting IgE-mediated MC activation as a therapeutic option for the treatment of food allergy, with particular focus on peanut allergy. However, two crucial preliminary considerations should be made. First, no treatment option currently available is uniquely targeting MCs. In fact, receptors inhibiting MC activation are shared among different cell types, and cytokines and other soluble mediators have pleiotropic effects affecting multiple cell populations at once. Second, any treatment inhibiting IgE-mediated MC activation should also take into consideration the broader implications and the potential loss of MC protective functions. Hence a benefit/risk assessment should be made, especially when considering highly disruptive interventions, like active MC depletion, not covered by the present manuscript (37).

## Allergen-Independent Approaches

### IgE-Mediated Mast Cell Activation

IgE antibodies are the mainstay of allergic responses. They are monomeric glycoproteins composed of two light and heavy chains, the latter showing four constant Ig-like domains (Cϵ1–4), bound *via* disulfide bridges (38). Several factors are involved in the development of functional IgE antibodies, including specific affinity maturation, conformational/allosteric properties, and glycosylation patterns (38–40). IgE blood concentration in healthy individuals is very low (below 210  IU/ml) compared to normal levels of IgG (5.65–17.65 mg/ml) (41, 42). IgE are mostly sequestered in peripheral tissues, with an average half-life estimated of 16–20 days in the skin versus 2–4 days in blood (43). Given the high affinity of FcϵRI to IgE (Kd = 10^10^–10^11^ M^−1^) and the slow dissociation rate (44, 45), the majority of IgE are cell-bound to either FcϵRI or the low-affinity receptor FcϵRII (CD23) *via* the Cϵ3-4 Fc domains (46). FcϵRI is the high-affinity IgE receptor constitutively expressed on MCs and basophils, while inducible on monocytes, dendritic cells, eosinophils and neutrophils (47–50). A tight correlation between atopic status, circulating IgE levels and surface expression of FcϵRI on MCs, basophils and other cell types has been proven (45, 47, 51, 52). While peripheral blood-resident cells acquire IgE directly from the circulation, perivascular tissue-resident MCs, sensing changes in total IgE levels, probe IgE from blood vessels using endoluminal cell processes (53).

Furthermore, occupancy of the FcϵRI receptor is crucial to ensure its expression on the cell membrane by MCs and basophils, as shown by mechanistic studies demonstrating increased FcϵRI expression upon IgE binding due to decreased FcϵRI endocytosis and degradation (44, 54–56). IgE bound to FcϵRI persists as long as MCs are alive, thus indicating that MCs preferentially display rather than catabolize IgE. FcϵRI-mediated constitutive internalization of IgE by dendritic cells and monocytes promotes serum IgE clearance instead (57).

FcϵRI is constituted by one alpha and one beta chain on MCs and basophils, or a single alpha chain on monocytes and dendritic cells (45, 58, 59), complexed with two additional gamma chains with immunoreceptor tyrosine-based activation motif (ITAM) domains acting as docking and activation sites for the Spleen tyrosine kinase (Syk) pathway (60, 61). The activation of the Syk, phosphatidylinositol 3-OH kinase/protein kinase B (PI3K/Akt) and extracellular signal-regulated kinase (ERK) pathways leads to increased intracellular calcium flux, calcium-dependent release of preformed mediators stored in intracellular granules and activation of transcription factors for eicosanoids, cytokines and chemokines production (62).

MCs and basophils express the highest density of FcϵRI receptor [estimated 0.7 × 10^5^ molecules per cell measured on LAD2 MCs (63)], with a bell-shaped dose–response when exposed to increasing allergen concentrations (64). Degranulation is tightly regulated *via* mechanisms modulating the MC activation threshold, not limited to IgE–FcϵRI complex expression. In fact, the nature and dose of the eliciting allergen also play a modulatory role. For instance, simultaneous stimulation using multiple allergens shows an additive effect on MC activation when suboptimal allergen concentrations are used. Conversely, stimulation with supra-optimal allergen concentrations inhibits MC degranulation (64, 65).

### Anti-IgE/FcϵRI Strategies

**Figure 1:**
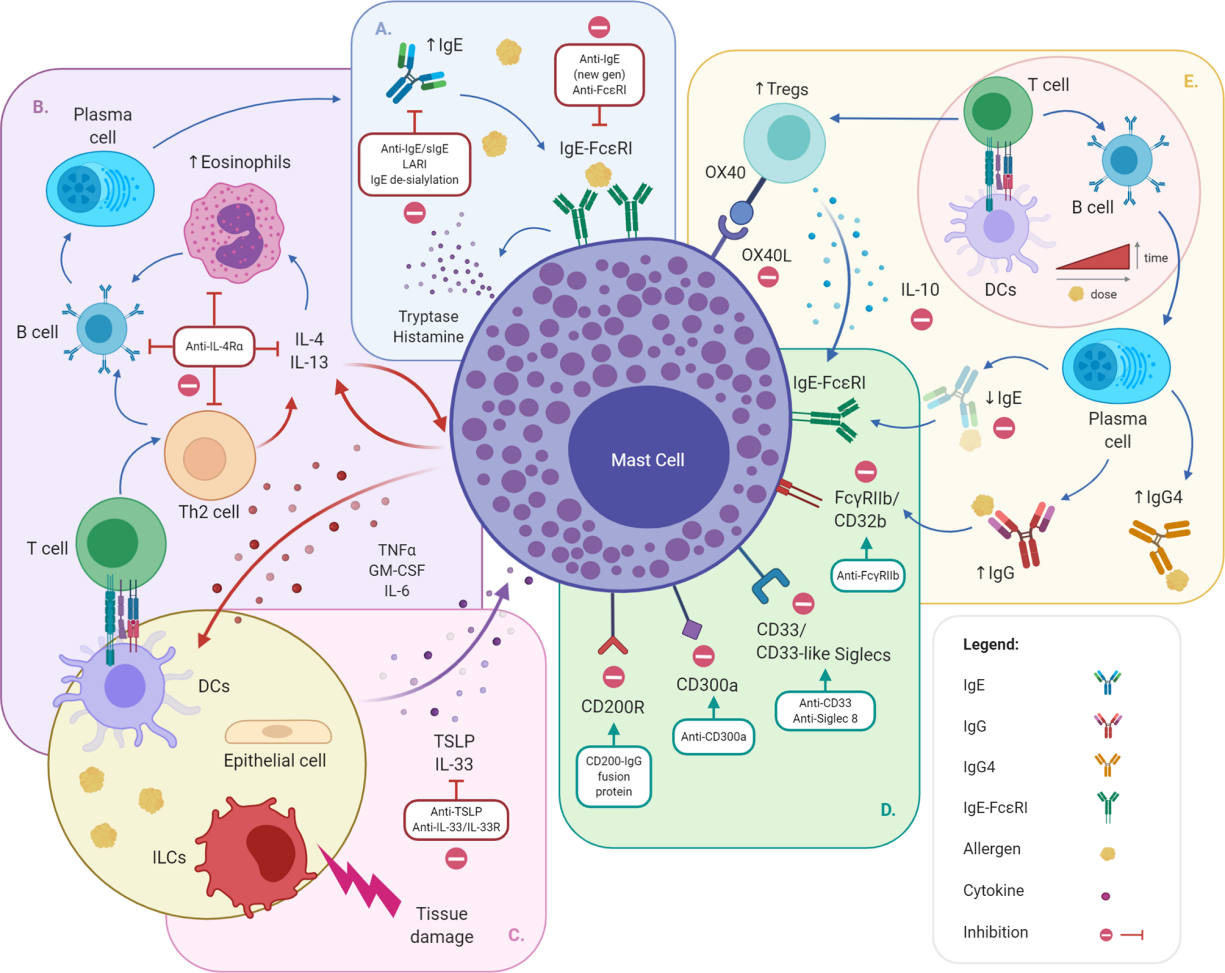
Approaches to target mast cell-dependent allergic responses. Summary of mechanisms controlling MC activation and degranulation and targeted inhibitory approaches, namely suppression of the IgE/FcϵRI axis **(A)**, modulation of IL-4/IL-13 **(B)** and IL-33/TSLP **(C)** cytokines, engagement of MC inhibitory receptors **(D)** and allergen immunotherapy **(E)**. DCs, dendritic cells; FcϵRI, high-affinity IgE receptor; FcγRIIb, low affinity IgG receptor b; IgE, immunoglobulin E; IgG, immunoglobulin G; IgG4, immunoglobulin G 4; IL-4Rα, interleukin 4 receptor alpha chain; LARI, low affinity allergic response inhibitors; MC, mast cell; Siglec, Sialic acid-binding immunoglobulin-type lectins; Tregs, T regulatory cells; TSLP, Thymic stromal lymphopoietin. Created with BioRender.com.

Given the pivotal role of IgE in the initiation and maintenance of allergic responses, increasing evidence supports the use of anti-IgE molecules as therapeutic strategy to treat allergic diseases, including food allergy (**Tables 1** and **2**). Anti-IgE therapy disrupts the IgE–FcϵRI axis *via* the active removal of circulating IgE and the downregulation of FcϵRI on MCs, basophils and dendritic cells (136–138). By removing circulating IgE, the turnover between circulating and cell-bound allergen-specific IgE (sIgE) slowly declines, ultimately reducing the amount of sIgE bound on the cell surface and decreasing the likelihood of allergen-IgE cross-linking and allergen-specific effector cell responses (139–141) (**Figure 1A**).

**Table 1:** List of clinical trials on allergen-dependent and -independent investigational treatments for peanut allergy.

	Strategy	Reference	Trial identifier	Study acronym	Investigational product	Phase	Placebo controlled	Age range	Tested peanut dose	Study status(as of 12/2020)
***Allergen-dependent treatments***	**EPI**	(66)	**NCT01170286**	PEP01.09	Epidermal Patch (peanut DBV712)	1	yes	6-50	20-100-250-500 mcg	
		(67, 68)	**NCT01904604**	DAIT CoFAR6	Epidermal Patch (peanut DBV712)	2	yes	4-25	100-250 mcg	
		(69)	**NCT01675882**	VIPES	Epidermal Patch (peanut DBV712)	2	yes	6-55	50 mcg	
		(70)	**NCT01955109**	OLFUS-VIPES	Epidermal Patch (peanut DBV712)	2	no	7-56	250 mcg	
		(71, 72)	**NCT02636699**	PEPITES	Epidermal Patch (peanut DBV712)	3	yes	4-11	250 mcg	
			**NCT02916446**	REALISE	Epidermal Patch (peanut DBV712)	3	yes	4-11	250 mcg	
			**NCT03211247**	EPITOPE	Epidermal Patch (peanut DBV712)	3	yes	1-3	250 mcg	
	**OIT**	(73)	**NCT01259804**	STOP-I	Peanut Flour	1	no	7-17	800 mg	
			**NCT02203799**	PeanutFlour	Peanut Flour	1	no	5-16	6-10 gr	
			**NCT01601522**	REB 07-348	Peanut Protein	1	yes	5-10	500 mg	
			**NCT04163562**	INP20-01	Peanut Oral Formulation (INP20)	1-2	yes	12-65	n/d	
		(74, 75)	**NCT00815035**	PnOIT3	Peanut Flour	2	yes	1-6	4-5-6 gr	
		(76)	**NCT00932828**	DEVIL	Peanut Flour	2	no	9-36	5 gr	
		(77–79)	**NCT02103270**	POISED	Peanut Protein	2	yes	7-55	300-4000 mg	
			**NCT01867671**	IMPACT	Liquid Extract, Peanut Flour	2	yes	12-48	5 gr	
			**NCT00597675**	PMIT	Peanut Flour	2	yes	1-18	4710 mg	
			**NCT03907397**	CAFETERIA	Peanut Protein	2	no	4-14	9043 mg	
		(80)	**NCT02046083**	PITA 3	Whole Peanuts (crushed)	2-3	yes	12-18	2 gr	
		(81)	**NCT02635776**	PALISADE	Peanut protein capsule (AR101)	3*	yes	4-55	1043 mg	
		(82)	**NCT03201003**	ARTEMIS	Peanut protein capsule (AR101)	3*	yes	4-17	2043 mg	
			**NCT03736447**	POSEIDON	Peanut protein capsule (AR101)	3*	yes	1-3	600-1000 mg	
		(83)	**n/d**	n/d	Whole Peanuts (crushed)	Other	no	3-14	500 mg	
		(84)	**n/d**	n/d	Peanut Flour	Other	yes	1-16	6 mg	
		(85)	**ISRCTN62416244**	STOP-II	Peanut Flour	Other	yes	7-16	1400 mg	
		(86)	**NCT02350660**	15098	Peanut Flour	Other	no	4-80	306 mg	
		(87)	**DRKS00004553**	Peanut OIT	Peanut Protein	Other	yes	3-17	300 mg	
		(88)	**NCT02457416**	TAKE-AWAY	Peanut Protein	Other	no	5-15	250-4000 mg	
			**NCT02149719**	BOPI-1	Boiled Peanut	Other	no	8-16	1400 mg	
			**NCT03937726**	BOPI-2	Boiled Peanut	Other	no	7-18	1440 mg	
			**NCT03532360**	2017-3204	Whole Peanuts (crushed)	Other	no	2-40	30-300-4172 mg	
			**NCT03648320**	GUPI	Peanut Protein	Other	no	18-40	1400 mg	
			**NCT04511494**	SmaChO	Peanut Protein	Other	no	1-3	775 mg	
	**OIT/SLIT**	(89)	**NCT01084174**	NA_00032256	Peanut Flour, Peanut Extract	1-2	yes	6-21	3.7 mg (SLIT), 2 gr (OIT)	
	**SLIT**		**NCT03070561**	JHU NA_00072576	Major Peanut Allergen Ara h 2 in Dissolving Film	Early 1	no	3-30	60 mcg	
			**NCT04603300**	INT301-101	Peanut Extract Toothpaste Formulation (INT301)	1	yes	18-55	n/d	
			**NCT03463135**	TDR14287	Glucopyranosyl Lipid A Peanut Extract	1	yes	12-55	n/d	
		(90, 91)	**NCT00580606**	DAIT CoFAR4	Glycerinated Allergenic Peanut Extract	1-2	yes	12-40	5 gr	
		(92)	**NCT01373242**	SLIT-TLC	Liquid Peanut Protein Extract	1-2	no	1-12	5 gr	
		(93–95)	**NCT00597727**	SLB	Liquid Peanut Protein Extract	2	yes	1-11	5 gr	
			**NCT02304991**	FARE/SLIT	Liquid Peanut Protein Extract	2	yes	12-48	5 mg	
			**NCT00429429**	1R21AT002557-02	Liquid Peanut Protein Extract	Other	no	6-35	8 gr	
	**SCIT/Vaccine**	(96)	**NCT00850668**	DAIT CoFAR1	E. Coli-Encapsulated, Recombinant Modified Peanut Proteins Ara h 1, Ara h 2, and Ara h 2 (EMP-123)	1	no	18-50	n/d	
			**NCT02163018**	HAL-MPE1/0043	Aluminium hydroxide adsorbed peanut extract (HAL-MPE1)	1	yes	18-65	n/d	
			**NCT02991885**	HAL-MPE1/0049	Aluminium hydroxide adsorbed peanut extract (HAL-MPE1)	1	yes	5-50	n/d	
			**NCT02851277**	0892-CL-1001	ARA-LAMP-vax (ASP0892), Multivalent Peanut (Ara h1, h2, h3) Lysosomal Associated Membrane Protein DNA Plasmid Vaccine	1	yes	18-55	n/d	
			**NCT03755713**	0892-CL-1002	ARA-LAMP-vax (ASP0892), Multivalent Peanut (Ara h1, h2, h3) Lysosomal Associated Membrane Protein DNA Plasmid Vaccine	1	yes	12-17	n/d	
			**NCT04200989**	IRB-19-7380	Intralymphatic Immunotherapy with Peanut Allergen	1-2	no	15-80	n/d	
	**Biologics + OIT**	(97–99)	**NCT01290913**	Xolair and Peanut Allergy	Omalizumab, Peanut Flour	1-2	no	7-25	1 gr	
		(100, 101)	**NCT02402231**	FASTXP201	Omalizumab, Peanut Flour	2	no	12-22	2800 mg	
			**NCT00932282**	PAIE/Xolair	Omalizumab, Peanut Flour	1-2	no	12+	950 mg - 20 gr	
			**NCT01781637**	PRROTECT	Omalizumab, Peanut Flour	1-2	yes	7-25	4 gr	
		(102)	**NCT01510626**	22872	Omalizumab, Multi-Allergen OIT	1	no	4-55	2 gr	
		(103)	**NCT02626611**	M-TAX	Omalizumab, Multi-Allergen OIT	2	yes	4-55	2 gr	
		(104)	**NCT04045301**	BOOM	Omalizumab, Multi-Allergen OIT	2	yes	6-25	1.5 gr	
			**NCT03881696**	OUtMATCH	Omalizumab, Multi-Allergen OIT	3	yes	1-55	600 mg	
			**NCT03682770**	R668-ALG-16114	Dupilumab, Peanut protein capsule (AR101)	2	yes	6-17	2044 mg	
***Allergen-independent treatments***	**Biologics**	(105)	**NCT00949078**	NA_00026397	Omalizumab	2	no	18-50	n/d	
		(106)	**NCT02643862**	MAP-X	Omalizumab	1-2	yes	4-55	2 gr	
			**NCT00382148**	Q3623g	Omalizumab	2	no	6-75	n/d	
			**NCT00086606**	Q2788g	Omalizumab	2	no	6-75	n/d	
			**NCT03679676**	IRB-47935	Omalizumab, Dupilumab	2	yes	6-25	1043 mg	
			**NCT03793608**	R668-ALG-1702	Dupilumab	2	no	6-17	n/d	
		(107)	**NCT02920021**	ANB020-003	ANB020 (Etokimab)	2	yes	18+	n/d	

*Phase 3 pivot trials only. EPI, Epicutaneous immunotherapy; n/d, not disclosed; OIT, Oral Immunotherapy; SLIT, Sublingual Immunotherapy.

◼ Completed ◼ Active, not recruiting ◼ Recruiting ◼ Not yet recruiting ◼ Terminated.

**Table 2:** List of clinical trials on biologics targeting key mechanisms of MC activation for conditions other than peanut allergy.

Biological target	Reference	Trial identifier	Study acronym	Investigational product	Condition(s)	Phase	Placebo controlled	Age range	Study status(as 12/2020)
**IgE**	(108)	**n/d**	n/d	Omalizumab	Asthma	3*	yes	12-75	
	(109)	**n/d**	n/d	Omalizumab	Asthma	3*	yes	12-76	
	(110)	**n/d**	n/d	Omalizumab	Asthma	3*	yes	12-75	
	(111)	**NCT00046748**	INNOVATE	Omalizumab	Asthma	3*	yes	12-75	
	(112)	**n/d**	SOLAR	Omalizumab	Asthma, Allergic Rhinitis	3*	yes	12-74	
	(113)	**NCT00314574**	EXTRA	Omalizumab	Asthma	3*	yes	12-75	
	(114)	**NCT00079937**	CIGE025AIA05	Omalizumab	Asthma	3*	yes	6-12	
	(115)	**NCT01287117**	ASTERIA I	Omalizumab	Chronic Spontaneous Urticaria	3*	yes	12-75	
	(116)	**NCT01292473**	ASTERIA II	Omalizumab	Chronic Spontaneous Urticaria	3*	yes	12-75	
	(117)	**NCT01264939**	GLACIAL	Omalizumab	Chronic Spontaneous Urticaria	3*	yes	12-75	
	(118)	**NCT03280550**	POLYP1	Omalizumab	Chronic Rhinosinusitis with Nasal Polyps	3*	yes	18-75	
	(118)	**NCT03280537**	POLYP2	Omalizumab	Chronic Rhinosinusitis with Nasal Polyps	3*	yes	18-75	
	(119–121)	**NCT00078195**	DAIT ITN019AD	Omalizumab, Ragweed AIT	Allergic Rhino-conjunctivitis, Grass Pollen Allergy	3	yes	18-50	
	(122)	**UMIN000015545**	n/d	Omalizumab, Cow's milk AIT	Cow's milk allergy	2	no	6-14	
	(84)	**NCT01157117**	DAIT AADCRC-MSSM-01	Omalizumab, Cow's milk AIT	Cow's milk allergy	2	yes	7-35	
		**NCT01703312**	CQGE031B2203	QGE031 (Ligelizumab)	Allergic Asthma	1-2	yes	18-65	
		**NCT01716754**	CQGE031B2201	QGE031 (Ligelizumab)	Asthma	2	yes	18-75	
		**NCT02336425**	CQGE031B2204	QGE031 (Ligelizumab)	Asthma	2	yes	18-75	
		**NCT01552629**	CQGE031X2201	QGE031 (Ligelizumab)	Atopic Dermatitis	2	yes	18-65	
		**NCT04513548**	CQGE031C2203	QGE031 (Ligelizumab)	Chronic Spontaneous Urticaria, Cholinergic Urticaria, Cold Urticaria	1	yes	18-79	
	(123)	**NCT02477332**	CQGE031C2201	QGE031 (Ligelizumab)	Chronic Spontaneous Urticaria	2	yes	18-75	
		**NCT03437278**	CQGE031C2202	QGE031 (Ligelizumab)	Chronic Spontaneous Urticaria	2	yes	12-18	
		**NCT03580369**	CQGE031C2302	QGE031 (Ligelizumab)	Chronic Spontaneous Urticaria	3	yes	12+	
		**NCT03580356**	CQGE031C2303	QGE031 (Ligelizumab)	Chronic Spontaneous Urticaria	3	yes	12+	
		**NCT01723254**	ANTI-IGE VACCINE	Anti-IgE Vaccine (PF-06444753, PF-06444752)	Allergic Rhinits	1	yes	18-55	
**IL-4Rα**		**NCT04442269**	R668-ABPA-1923	Dupilumab	Allergic Bronchopulmonary Aspergillosis	3	yes	12+	
		**NCT03935971**	2018P002882	Dupilumab	Allergic Contact Dermatitis	4	no	18+	
		**NCT03558997**	R668-ALG-16115	Dupilumab	Allergic Rhinoconjunctivitis, Grass Pollen Allergy	2	yes	18-55	
		**NCT04502966**	GRADUATE	Dupilumab	Allergic Rhinoconjunctivitis, Grass Pollen Allergy	2	yes	18-65	
		**NCT03595488**	1828-A-18	Dupilumab	Aspirin-exacerbated Respiratory Disease	2	no	18+	
		**NCT04442256**	2019-004889-18	Dupilumab	Aspirin-exacerbated Respiratory Disease	4	no	18-70	
	(124)	**NCT02528214**	VENTURE	Dupilumab	Asthma	3*	yes	12+	
	(125, 126)	**NCT02414854**	Liberty Asthma Quest	Dupilumab	Asthma	3*	yes	12+	
		**NCT03560466**	Liberty Asthma Excursion	Dupilumab	Asthma	3*	no	7-12	
		**NCT02948959**	VOYAGE	Dupilumab	Asthma	3*	yes	6-11	
		**NCT03782532**	EFC13995	Dupilumab	Asthma	3*	yes	12+	
	(127)	**NCT02260986**	CHRONOS	Dupilumab	Atopic Dermatitis	3*	yes	18+	
	(128)	**NCT02277743**	SOLO 1	Dupilumab	Atopic Dermatitis	3*	yes	18+	
	(128)	**NCT02277769**	SOLO 2	Dupilumab	Atopic Dermatitis	3*	yes	18+	
	(129)	**NCT02395133**	SOLO-CONTINUE	Dupilumab	Atopic Dermatitis	3*	yes	18+	
	(130, 131)	**NCT03054428**	R668-AD-1526	Dupilumab	Atopic Dermatitis	3*	yes	12-17	
		**NCT03346434**	Liberty AD	Dupilumab	Atopic Dermatitis	2-3	yes	6 mo-5	
		**NCT04296864**	18-290-0002	Dupilumab	Atopic Keratoconjunctivitis	2	no	18+	
		**NCT03749148**	CHED	Dupilumab	Cholinergic Urticaria	2	yes	18-75	
	(132)	**NCT02912468**	SINUS-24	Dupilumab	Chronic Rhinosinusitis with Nasal Polyps	3*	yes	18+	
	(132)	**NCT02898454**	SINUS-52	Dupilumab	Chronic Rhinosinusitis with Nasal Polyps	3*	yes	18+	
		**NCT04362501**	IRB00229130	Dupilumab	Chronic Rhinosinusitis without Nasal Polyps	2	yes	18-75	
		**NCT04180488**	CUPID	Dupilumab	Chronic Spontaneous Urticaria	3	yes	6-80	
		**NCT03749135**	DUPICSU	Dupilumab	Chronic Spontaneous Urticaria	2	yes	18-75	
	(133)	**NCT02379052**	R668-EE-1324	Dupilumab	Eosinophilic Esophagitis	2	yes	18-65	
		**NCT03633617**	R668-EE-1774	Dupilumab	Eosinophilic Esophagitis	3	yes	12+	
		**NCT04394351**	EoE KIDS	Dupilumab	Eosinophilic Esophagitis	3	yes	1-11	
		**NCT03678545**	IRB 2018-4246	Dupilumab	Eosinophilic Gastroenteritis	2	yes	12-70	
		**NCT04148352**	IRB-52976	Dupilumab	Milk Allergy	2	yes	4-50	
		**NCT04430179**	STUDY000808	Dupilumab	Severe Eosinophilic Chronic Sinusitis	2	yes	18-65	
**IL-33**		**NCT03533751**	ATLAS	ANB020 (Etokimab)	Atopic Dermatitis	2	yes	18-75	
		**NCT03614923**	ANB020-006	ANB020 (Etokimab)	Chronic Rhinosinusitis	2	yes	18-70	
		**NCT03469934**	ANB020-004	ANB020 (Etokimab)	Eosinophilic Asthma	2	yes	18-65	
**ST2/IL-33R**		**NCT03615040**	COPD-ST2OP	MSTT1041A	Chronic Obstructive Pulmonary Disease	2	yes	40+	
**TSLP**	(134)	**NCT01405963**	20101183	AMG 157 (Tezepelumab)	Asthma	1	yes	18-60	
		**NCT02698501**	UPSTREAM	MEDI9929 (Tezepelumab)	Asthma	2	yes	18-75	
		**NCT02237196**	CATNIP	AMG 157 (Tezepelumab), Cat AIT	Cat Allergy, Cat Hypersensitivity	1-2	yes	18-65	
**Siglec 8**		**NCT03379311**	KRONOS	AK002 (Lirentelimab)	Atopic Keratoconjunctivitis	1	no	18-80	
		**NCT03436797**	CURSIG	AK002 (Lirentelimab)	Chronic Spontaneous Urticaria	2	no	18-85	
	(135)	**NCT03496571**	ENIGMA	AK002 (Lirentelimab)	Eosinophilic Gastroenteritis	2	yes	18-80	
		**NCT03664960**	AK002-003X	AK002 (Lirentelimab)	Eosinophilic Gastroenteritis	2	no	18-80	
		**NCT04322708**	KRYPTOS	AK002 (Lirentelimab)	Eosinophilic Esophagitis	2-3	yes	12-80	
		**NCT04322604**	ENIGMA 2	AK002 (Lirentelimab)	Eosinophilic Gastroenteritis	3	yes	18-80	
		**NCT02808793**	AK002-001	AK002 (Lirentelimab)	Indolent Systemic Mastocytosis	1	no	18-65	

*Phase 3 pivot trials only. AIT, Allergen immunotherapy; n/d, not disclosed.

◼ Completed ◼ Active, not recruiting ◼ Recruiting ◼ Not yet recruiting ◼ Terminated.

Furthermore, anti-IgE treatment induces FcϵRI down-regulation by interfering with the accumulation of IgE–FcϵRI complexes occurring at the cell surface due to reduced receptor occupancy by IgE (54–56, 136). The reduced availability of sIgE–FcϵRI complexes further inhibits the release of Th2 cytokines and allergic mediators upon allergen challenge by MCs, basophils and dendritic cells (136, 137, 141–144).

Some anti-IgE treatments also inhibit IgE binding to the CD23 receptor, the low affinity IgE receptor constitutively expressed on naïve B cells, exerting an inhibitory effect on IgE-mediated antigen presentation (145, 146), inducing anergy or apoptosis of membrane IgE-bearing B cells (147, 148) and in some cases modulating IgE production (146, 149). However, treatment discontinuation is followed by the quick restoration of pre-treatment IgE levels (150).

Omalizumab, a humanized anti-IgE monoclonal antibody, is the first and most studied biologic, currently used to treat severe asthma and chronic spontaneous urticaria (**Table 2**). It binds to IgE Cε3 domains, outside of the FcεRI-binding site, and sterically disrupts binding to both FcϵRI and CD23 (151). Omalizumab does not affect pre-bound IgE-receptor interactions, due to conformational changes of receptor-bound IgE masking omalizumab binding sites, and does not induce IgE cross-linking on the cell surface (151, 152).

Omalizumab downregulates the surface expression of FcϵRI in both basophils and MCs (153). However, while FcϵRI expression declines rapidly in circulating basophils (less than 24 h), this process requires longer time in tissue resident MCs (estimated 10–20 days) (136, 154, 155).

The effects exerted by omalizumab on MCs are of clinical relevance also in non-IgE-mediated diseases such as inducible urticarias (156) and MC activation syndrome (105), thus suggesting a broad MC stabilizing function. In food allergy, several clinical trials and real-life evidence showed the safety and usefulness in inhibiting allergic responses of omalizumab as monotherapy (157–160) (**Table 1**), or in association with allergen-specific immunotherapy, further discussed in *Combination Treatments With Biologics* section.

Designed Ankyrin Repeat Proteins (DARPins), genetically engineered antibody mimetic proteins, recognize IgE Cϵ3 domains with high specificity and affinity, and have been shown to be 10,000-fold more efficient than omalizumab in dissociating IgE complexes *in vitro* and in both *ex vivo* transgenic mouse models and human tissues. Thus, their rapid onset of action makes them of particular interest as treatment option to thwart pre-initiated anaphylaxis episodes (161–165) (**Table 3**). Along with DARPins, other new generation high-affinity anti-IgE monoclonal antibodies like ligelizumab can actively bind IgE Cε3–4 fragments and efficiently disrupt IgE-FcεRI complexes without, however, interfering with CD23 binding, differently than omalizumab (146) (**Table 3**). Thus, DARPins and ligelizumab might improve treatment efficacy in food allergy, albeit to date no trials on food allergy are ongoing (**Table 2**).

**Table 3:** Interventions aimed at reducing IgE-mediated mast cell activation currently at pre-clinical/early clinical stage.

Biological target	Reference	Intervention	Observed results	Food allergens tested	Human tested*	Experimental setup
**IgE**	(162)	**DARPin E2_79 (E001)**	E001 binds to IgE-Cϵ3 domains, promoting active disassociation of pre-formed IgE-FcϵRI complexes *via* allosteric inhibition	no	no	Selection of DARPins and surface plasmon resonance, fluorescence and ELISA binding assays *in vitro*
	(163)	**DARPin E2_79 (E001)**	E001 binds to IgE-Cϵ3 domains, promoting active disassociation of pre-formed IgE-FcϵRI complexes *via* allosteric inhibition	no	yes	Selection of DARPins, analysis of recombinant proteins in ELISA and surface plasmon resonance
		**Biparatopic DARPin bi53_79 (E002)**	E002 is a biparatopic variant complexing E001 to a second anti-IgE (DARPin E3_53) recognizing receptor-bound IgE, showing higher disruptive efficacy on IgE-FcϵRI complexes			Human primary basophils FcϵRI expression and degranulation assays FcϵRIα-chain transgenic mice for passive cutaneous anaphylaxis test
	(164)	**Biparatopic DARPin bi53_79 (E002)**	E002 binds to IgE-Cϵ3 domains and receptor-bound IgE, actively disrupting IgE-FcϵRI complexes	no	yes	Culture of human PCLS sensitized with plasma of HDM-allergic donors
						Lung mast cell histamine release and bronchoconstriction after challenge with HDM
	(165)	**Trivalent DARPins (KIH_E07_79, tri11_53_79, tri11_E07_79)**	Rapid disassociation of pre-formed IgE-FcϵRI complexes inhibits degranulation and terminates pre-initiated allergic reactions. Co-engagement of FcγRIIb receptor improves the disruptive efficacy and reduces anaphylactogenicity.	no	yes	Isolated human basophils sensitized to grass pollen mix huIgE/huFcϵRIα^dtg^ transgenic mice sensitized to Vitamin D analogue MC903 plus OVA for PCA, NIP_20_ for PSA
	(40)	**De-sialylation of IgEs**	Removal of sialic acid residues from IgEs of allergic donors attenuates degranulation by effector cells and reduces anaphylaxis	peanut	yes	De-sialylation of IgE using neuroaminidase fusion protein (NEU^Fcϵ^)
						Human BAT and LAD2 MC degranulation assay using peanut, birch tree pollen, HDM, cat allergic and non-allergic sera before and after de-sialylation
						BALB/c OVA PCA mouse model
	(166)	**Peptide-based anti-IgE conjugate vaccine**	Vaccine using virus-like particles conjugated to peptides and adjuvants to generate antibodies binding to the IgE Cϵ3 domain, promoting the active removal of circulating IgE	no	yes	Quantification of serum IgE levels pre and post treatment in Cynomolgus monkeys, competition ELISA for anti-IgE antibody avidity testing with human sera
	(167)	**Self-assembled peptide-based anti-IgE vaccine**	Vaccine using self-assembled peptides to generate antibodies binding to the IgE Cϵ3 domain, promoting the active removal of circulating IgE and inhibition of acute IgE-mediated anaphylaxis	no	no	CD-1 mice DNP anaphylaxis model, quantification of mouse free IgE levels *via* competition ELISA
**sIgE**	(168)	**Covalent Heterobivalent Inhibitors (cHBIs)**	Irreversible binding to circulating human sIgE specific for Ara h2 and Ara h 6	peanut	yes	Human BAT using Ara h 2 - Ara h 6 sera from peanut allergic patients with or without cHBIs
**FcϵRI**	(169)	**Anti-human FcϵRI monoclonal antibodies**	Binding to human FcϵRI, rapid suppression of IgE-mediated anaphylaxis and rapid desensitization achieved and maintained using repeated small doses. Treatment induces loss of blood basophils, removal of membrane IgE and FcϵRIα on mouse peritoneal MCs	egg	yes	huFcϵRIα/F709 expressing huFcϵRIα and huIL-4Rα anaphylaxis and desensitization model Immunodeficient reNSGS mice reconstituted with T cell-depleted human cord blood for the analysis of human basophils and MCs
**BTK**	(170)	**Ibrutinib, Acalabrutinib**	Inhibited IgE-mediated degranulation and release of IL-6, IL-8, IL-10, MCP-1 and GM-CSF by skin-derived human MCs. Prevented IgE-mediated bronchoconstriction and anaphylaxis	no	yes	Skin-derived human MCs, bronchial constriction assay using isolated human bronchi. PSA model using NSG-SGM3 humanized mice sensitized to NP
**IL-4Rα**	(171)	**Dupilumab, IL-4/IL-13 MC priming (indirect evidence of the effects of IL-4Rα blockade)**	Dupilumab prevents the expression of chemokines, proinflammatory Th2 cytokines and eosinophil infiltration in the lungs, while not affecting circulating eosinophils. Exposure to IL-4 enhances IgE-mediated MC responses, causing an increase in Th2-associated chemokine and cytokine gene expression upon IgE crosslinking	no	yes	Il4ra^hu/hu^ Il4^hu/hu^ mice lung inflammation model using intranasally administered IL-4 and IL-13 In vitro-generated human MCs cultured with or without IL-4, IL-13 and stimulated with Fel d 1- Fel d 1 IgE
**TSLP–IL-25–IL-33R/ST2**	(172)	**Anti-mouse TSLP, IL-25 and IL-33R/ST2 monoclonal antibody cocktail**	Binding and neutralization of key alarmins TSLP, IL-25 and IL-33 cytokine receptor. Suppression of established allergy and anaphylaxis upon allergen challenge, reduction and prevention of sensitization to allergens	egg	no	BALB/c mice medium-chain tryglicerides plus egg white anaphylaxis model Cytokine, antibodies and mouse mast cell protease 1 measurement by ELISA, immunofluorescence and flow cytometry for tissue analysis
**FcγRIIb**	(173)	**FcγRIIb–FcϵRIα bifunctional fusion protein**	Simultaneous binding of FcγRIIb and FcϵRIα inhibits Syk phosphorilation and FcϵRIα-mediated activation	no	yes	Binding analysis on CHO3D10 and HMC-1 cells expressing FcγRIIb
						Human basophil histamine release using NIP/anti-NIP stimulation
						Transgenic mice expressing human FcϵRIα NP PCA model
	(174)	**Anti-IgE/FcγRIIb fusion protein (bivalent DARPin E53 and DE53-Fc)**	Simultaneous binding to FcϵRI-bound IgE and FcγRIIb inhibits basophil and MC activation	no	yes	Selection of DARPins and surface plasmon resonance Human BAT using grass pollen extracts with/without DE53-Fc and bivalent DARPin E53
	(175)	**Ara h 2–Fcγ fusion protein (AHG2)**	Inhibition of peanut-specific anaphylaxis and inhibition of histamine release by engagement of FcγRIIb, decreased airways induced inflammation by peanut challenge	peanut	yes	Fluorescence binding assay to HMC-1 mast cell line Human basophil histamine release using whole peanut extracts
						Transgenic mice expressing human FcϵRIa and C57BL/6 and Fcgr2b^tmiTtk^ mice peanut allergy model
	(176)	**Anti-IgE/FcγRIIb fusion protein (D11_E53)**	Simultaneous binding to FcϵRI-bound IgE and FcγRIIb inhibits basophil degranulation and anaphylaxis, abrogating intracellular activation signaling pathways	no	yes	Selection of DARPins, surface plasmon resonance and ELISA binding assays Human primary basophils from healthy and grass pollen allergic donors used for BAT, inhibition assay
						Transgenic mice expressing human FcϵRIa anaphylaxis model
	(177)	**Anti-Ara h 2 monoclonal antibody**	Anti-Ara h 2 binds to FcγRIIb receptor, inhibits systemic and local allergic reactions elicited by peanut and protects from anaphylaxis	peanut	no	BALB/c mice sensitized intraperitoneally with peanut extract, local and intravenous anaphylaxis model
**CD200R**	(178)	**Soluble CD200-IgG fusion protein**	Inhibition of FcϵRI-mediated MC degranulation and cytokine secretion	no	yes	Human cord-blood derived and skin MCs, mouse C57BL/6 bone marrow and skin MCs
						MC degranulation assays using anti-FcϵRI monoclonal antibodies, cytokine assay by ELISA
**CD300a**	(179)	**Bispecific IgE-CD300a antibody fragment (IE1)**	Dose-dependent inhibition of signaling events induced by FcϵRI and IgE-mediated MC degranulation *in vitro*, abrogates anaphylaxis and allergic airway inflammation *in vitro*	no	yes	Human cord blood-derived MCs, Murine bone marrow-derived MCs BALB/c DNP PCA mouse model, OVA-sensitized asthma model
**Siglec 3 (CD33)**	(180)	**Liposomal nanoparticles coated with CD33L and antigen (TNP)**	Engagement of CD33 prevents antigen-specific degranulation, suppresses MC IgE-mediated activation and anaphylaxis and inhibits IgE-mediated airway bronchoconstriction *via* phosphorylation of Syk, PLCγ1, MEK and ERK	peanut	yes	Human LAD2 and skin-derived MCs
						Lung PCLS bronchoconstriction challenge
						Humanized Mcpt5-Cre^+/–^R26-CD33+ TNP PCA and PSA mouse models, peritoneal MCs
**Siglec 8**	(181)	**Anti-Siglec 8 monoclonal antibodies**	Engagement of Siglec-8 on MCs inhibits FcϵRI-dependent release of mediators, except IL-8, reduces calcium flux and anti-IgE-evoked bronchoconstriction	no	yes	Human CD34-derived MCs Intrapulmonary bronchi for bronchoconstriction challenge using anti-IgE
						RBL- 2H3 cells transfected with normal and mutated forms of Siglec-8
	(182)	**AK002 (lirentelimab)**	AK002 induced apoptosis of eosinophils activated with IL-5, promoted antibody-dependant cell cytotoxicity by NK cells, reduced the infiltration of eosinophils in lung tissues and prevented anaphylaxis through the inhibition of MCs	no	yes	Human peripheral blood eosinophils and lung tissues
						NSG-SGM3 BLT mice NP PSA model
	(135)	**AK002 (lirentelimab)**	AK002 decreases eosinophils in sputum and inhibits IgE-mediated activation of MCs in lung tissues	no	yes	Sputum and lung tissue from asthma patients, analysis of gene expression for eosinophils and MCs, MC activation assay using anti-FcϵRI antibodies
**Other**	(183)	**Intranasal casein nanoemulsion vaccine**	Suppression of MC activation and infiltration in small intestine upon oral challenge. Broad reduction in Th2 immunity against casein, increased Th1, Th17 and IL-10 responses.	cow’s milk	no	BALB/c mice sensitized to casein and intranasally immunized using casein mixed with 20% nanoemulsion adjuvant (ultra‐pure soybean oil with cetylpyridinium chloride). Duodenal and jejunal MCs quantification *via* tissue sections
	(184)	**Vaccine using engineered virus-like particles displaying major peanut allergens (CuMVtt-Ara R, CuMVtt-Ara h 1, CuMVtt-Ara h 2)**	Protection against anaphylaxis, induction of peanut-specific IgG antibodies, reduced tissue infiltration by eosinophils and MCs, reduced MC activation upon allergen challenge	peanut	no	BALB/c mice peanut anaphylaxis model, subcutaneous immunization with CuMVtt combined with either whole extract of roasted peanut (Ara R), Ara h 1 or Ara h 2 Murine bone marrow–derived MCs sensitized with sera of mice sensitized to peanut and challenged with peanut extract

*Tested in human sera/cells/tissues. BAT, basophil activation test; BTK, Bruton Tyrosine Kinase, CuMVtt, Cucumber Mosaic Virus including tetanus toxin epitopes; DARPin, Designed Ankyrin Repeat Protein; DNP, dinitrophenol; ELISA, Enzyme-linked Immunosorbent Assay; FcϵRI, high-affinity IgE receptor 1; FcγRIIb, Fc gamma receptor II b; HDM, house dust mites; IgE, Immunoglobulin E; IL-4Rα, Interleukin-4 receptor alpha; IL-33R/ST2, Interleukin -33 receptor; MC, mast cell; NIP, 4-hydroxy-3-iodo-5-nitrophenylacetyl; NK, natural killer; NP, 4-hydroxy-3-nitrophenylacetyl; OVA, ovalbumin; PCA, passive cutaneous anaphylaxis; PCLS, precision-cut lung slices; PSA, passive systemic anaphylaxis; sIgE, allergen-specific immunoglobulin E; TNP, trinitrophenol; TSLP, thymic stromal lymphopoietin.

New anti-IgE strategies involve self-assembled mRNA vaccines, that provide epitopes mimicking IgE Cε3 domains and stimulate the production of endogenous anti-IgE IgG antibodies, eventually modulating circulating IgE levels *via* the same mechanisms of omalizumab and other anti-IgE molecules (166, 167). These new treatments inhibited IgE-mediated anaphylaxis in animal models (**Table 3**) and were tested in a Phase I trial conducted on allergic rhinitis patients (NCT01723254, **Table 2**); however their application in food allergy is still unclear.

Concerns over the long-lasting implications of irreversible IgE suppression might also arise, considering that, along with omalizumab and other high affinity molecules, broad anti-IgE agents bind also to IgE antibodies serving housekeeping functions, like protection against parasitic infections and tumor surveillance (185–187). Therefore, alternative strategies have been developed to specifically target IgE of interest. The use of covalent heterobivalent low affinity allergic response inhibitors (LARI) has been promising and showed to reduce the risk of anaphylaxis in experimental mouse models of peanut allergy (168) (**Table 3**).

Alternatively to anti-IgE molecules, a recent approach using anti-FcϵRIα monoclonal antibodies strongly suppressed IgE-mediated MC activation in a humanized mouse model of food allergy and anaphylaxis, revealing another promising therapeutic option (169) (**Table 3**).

Furthermore, inhibition of FcϵRI-mediated signaling using Bruton Tyrosin Kinase (BTK) inhibitors, significantly reduced degranulation and cytokine production in human MCs and basophils, decreased bronchoconstriction in isolated human bronchi, and proved effective in preventing anaphylaxis in a passive systemic anaphylaxis model using humanized mice (170, 188) (**Table 3**). Although ibrutinib is well known for its gastrointestinal, cardiovascular and hematological side effects, newer generation molecules, like acalabrutinib, show better safety profile and could become effective, fast-acting oral treatments (189). To this date, however, no clinical trials using BTK inhibitors in food allergy are on-going.

Current evidence suggests that IgE from atopic individuals show an increased sialic acid content, contrary to subjects with no atopy, thus pointing at an important role of sialylation to determine IgE allergenicity (40). Neuraminidase-induced de-sialylation of IgE in a non-FcϵRI dependent manner also diminished downstream signaling in MCs (40). Therefore, de-sialylation of IgE promises to decrease IgE allergenicity, without disrupting non-allergenic IgE activity (**Table 3**). However, sialidases are ubiquitously expressed in human tissues and play an important role in a variety of physiological and pathological processes, including tumor, infection and inflammation (190), hence the manipulation of the sialylation axis remains an ambitious goal. Notwithstanding, selective small molecule inhibitors of human sialidases hold a great potential for therapeutic development and warrants further investigation (191).

### Cytokines Modulating Mast Cell Activity in Allergy

Cytokines involved in Th2 responses, such as IL-4 and IL-13, promote MC proliferation, FcϵRI expression, IgE-mediated degranulation and cytokine production, adhesion and chemotaxis (171, 192, 193). IL-4 and IL-13 receptors share a common alpha chain (IL-4Rα), broadly expressed on lymphocytes, granulocytes and MCs, forming different functional heterodimers according to the associated beta chain (i.e. IL-4R Type I and II, IL-13R), which ultimately activate the intracellular signal transducer and activator of transcription 6 (STAT6) *via* the phosphorylation of Janus Kinases (Jak1-3, Tyk2) (194, 195). In particular, the proliferation and chemotaxis of MCs induced by IL-4/IL-4R engagement in mucosal interfaces are crucial for the amplification of local allergen responses and responsible for augmented permeability in the intestines and enhanced sensitivity to food allergens and anaphylaxis in experimental mouse models (196–198).

Alongside classical Th2 cytokines, MCs respond rapidly to tissue damage signals such as IL-33 and thymic stromal lymphopoietin (TSLP), alarmins produced mostly by epithelia, innate lymphoid cells and, in some conditions, by MCs themselves (199, 200). IL-33 is known to promote maturation and survival of MCs, enhance the production of pre-formed mediators (e.g. tryptase, serotonin) (201), cytokines (e.g. IL-4, IL-6, IL-13, GM-CSF), and chemokines (e.g. CCL2, CCL17) (201–203), while inhibiting the expression of regulatory cytokines, such as IL-10 (204). Furthermore, IL-33 potentiates IgE-mediated degranulation (202). However, a long-lasting IL-33 stimulation downregulates FcϵRI expression in human MCs, thus inhibiting IgE-dependent MC activation (201), and generating a hyporesponsive phenotype in both mouse and human MCs (205).

TSLP shares common properties with IL-33. They both promote the proliferation and differentiation of MC progenitors (206), and the production of pro-inflammatory cytokines (IL-5, IL-6, IL-13, GM-CSF) and chemokines (CXCL8, CCL1) without inducing the release of pre-formed granule mediators (207). In a food allergy mouse model, TSLP participates in the skin sensitization to food antigens, promoting basophil recruitment and initiating Th2 responses, whereas IL-33 is essential for gut-mediated sensitization and effector responses, including anaphylaxis (208).

### Anti-Cytokine Treatments (IL-4/13, IL-33, TSLP)

Several anti-cytokine treatments have shown promising results in food allergy. The monoclonal antibody dupilumab, blocking IL-4 and IL-13 from binding to the IL-4Rα chain, is currently approved for treatment of severe atopic dermatitis and asthma (**Table 2**). IL4Rα blockade broadly reduces Th2-responses (171) while increasing Treg suppressive responses (98), reduces eosinophil infiltration (171) and MC proliferation in mucosal tissues of IL4Rα^−/−^ mice (198). Dupilumab potentially inhibits MC priming and enhancement of IgE-mediated responses by IL-4 (171) (**Table 3**), while hampering B cell activation and IgE synthesis in mice (171, 209). In fact, recent evidence shows an important role of dupilumab in modulating B cell recall responses, as demonstrated by the reduction of peanut-specific IgE production by human B cells *in vitro*, and sustained inhibition after *in vivo* re-exposure in a peanut anaphylaxis mouse model (210) (**Figure 1B**). Albeit limited to a single case report, dupilumab is an efficient therapeutic option for multiple co-occurring food allergies (211), and under clinical trial as treatment for peanut allergy (**Table 1**).

The upstream role of IL-33 and TSLP in promoting Th2 responses makes them interesting targets for the treatment of atopic conditions, including food allergy (36) (**Figure 1C**). In a Phase II study 73% of peanut allergic patients treated with the anti-IL-33 antibody etokimab achieved tolerance to target peanut dose, showing reduced IL-4, IL-5, IL-13 and IL-9 production after an *in vitro* T cell challenge with peanut extract, along with reduced peanut-specific IgE levels compared to the placebo arm (107) (NCT02920021, **Table 1**). As for TSLP blockade, mouse models suggest some efficacy, in combination with either IL-25 or IL-33 receptor monoclonal antibodies, in preventing sensitization to food allergens, and promoting tolerance in association with oral immunotherapy (172) (**Table 3**). Anti-TSLP (tezepelumab, AMG 157, MEDI9929) has been successfully used in reducing allergen-induced bronchoconstriction and indexes of airway inflammation in patients with allergic asthma (NCT01405963) (134, 212) and is currently under investigation in a study combining tezepelumab with allergen-specific immunotherapy for the induction of tolerance in subjects with cat allergy (NCT02237196, **Table 2**). However, no clinical studies assessing the efficacy of anti-TSLP treatment in food allergy are currently on-going.

## Exploiting Mast Cell Inhibitory Receptors

Known inhibitory receptors of IgE-mediated MC activation are the Fc gamma receptor FcγRIIb, CD200R, Sialic acid-binding immunoglobulin-type lectins (Siglec) of the CD33 family and CD300a. Most inhibitory receptors exert broad suppressive functions on MC activation, with the exception of FcγRIIb and CD200R, producing allergen-specific inhibition.

Excluding CD200R, all inhibitory receptors expressed on MCs show intracellular immunoreceptor tyrosine-based inhibition motif (ITIM) domains that actively inhibit the phosphorylation of the Syk pathway *via* the recruitment of tyrosine phosphatases with Src homology 2 domains (e.g. SHP, Grb2 and SHIP), or PI3K binding-motifs (213, 214), disrupting intracellular calcium flux and IgE-dependent intracellular activation (**Figure 1D**).

FcγRII/CD32 receptors are immunoglobulin-like transmembrane proteins binding to the hinge region of IgG and IgG immune complexes. Of the three different subtypes, namely, FcγRIIa (CD32a), FcγRIIb (CD32b), and FcγRIIc (CD32c), only FcγRIIb is inhibitory. In mice, IgG binding to FcγRIIb inhibits antigen-specific IgE-mediated activation and Th2 cytokine production by MCs, IgE antibody production by B cells (215–218), while promoting dendritic cell-mediated mucosal tolerance by inducing Treg recruitment in the gut (215, 217, 218). In humans, while FcγRIIb is widely expressed on B cells, dendritic cells, monocytes and basophils (219), FcγRIIb transcripts are detectable in gastrointestinal MCs (220), but not skin MCs (221). Although the expression of FcγRIIb by gut MCs could correlate with increased pro-tolerogenic functions, the lack of FcγRIIb-mediated inhibition on skin MCs could be a reason for the increased risk of allergic sensitization *via* the skin compared to the gut route, as currently suggested by the dual exposure hypothesis (222), and diverging clinical responses observed in the skin versus gut after allergen immunotherapy (220).

Given the antigen-specific nature of FcγRIIb-mediated tolerance, its engagement could be especially useful to selectively inhibit food allergic reactions. Promising results have been achieved in *in vitro* studies using human basophils, bone marrow-derived MCs of human FcϵRIα-transgenic mice, FcϵRIα-transfected human cell lines and the HMC-1 mast cell line (173, 174, 176) (**Table 3**). Conversely, FcγRIIb bispecific molecules specifically targeted to major allergenic epitopes reduced allergen-specific responses in a peanut allergy mouse model using an Ara h 2-FcγRIIb fusion protein (175) (**Table 3**). Furthermore, FcγRIIb exerts a pivotal role in the generation of allergen-specific tolerance during the course of allergen immunotherapy, as outlined in *Modulation of Mast Cell Reactivity Using Allergen-Specific Immunotherapy*.

As member of the Immunoglobulin receptor superfamily, CD200R is an inhibitory receptor widely expressed on myeloid cells and skin MCs, shown to hinder MC activation and cytokine release in the absence of ITIM domains but in need of FcϵRI co-ligation, similar to FcγR receptors (178, 223). Antibodies targeting CD200R were effective in inhibiting MC activation in experimental mouse models and *in vitro* and tissue-derived human MCs (178) (**Table 3**), but no evidence of efficacy in food allergy models has been provided to date.

Siglec receptors selectively bind to sialic acid-containing glycoproteins, each with a specific sialoside ligand preference (224). Among the many Siglec receptors expressed by human MCs [i.e. Siglec 2, 3 (CD33), 5 through 10] (225, 226), CD33 and other CD33-like molecules (i.e. Siglec 5–11) are inhibitory receptors with intracellular ITIM/ITIM-like domains inhibiting FcϵRI-dependent activation (227, 228).

Beyond their suppressive role in IgE-mediated activation, recent evidence also suggests an inhibitory role in IL-33-mediated activation of MCs, with reduction of airway inflammation and fibrosis markers, studied in non-allergic mouse models of cigarette-induced chronic obstructive bronchopulmonary disease and bleomycin-induced lung injury (229).

Siglec 3 and 8 are currently the most promising targets in the treatment of allergic diseases. In fact, CD33 ligand-coated liposomal nanoparticles suppress MC activation, prevent IgE-mediated anaphylaxis and induce allergen desensitization lasting a few days in ovalbumin and peanut allergy mouse models (180) (**Table 3**). On the other hand, the engagement of Siglec 8 reduces intracellular calcium flux and FcϵRI-dependent release of mediators on human MCs (181, 229), while exerting a potent pro-apoptotic effect on human eosinophils and reducing tissue distribution *ex vivo* (135, 182, 230) (**Table 3**). Furthermore, in a humanized mouse model, lirentelimab (AK002) successfully inhibited IgE-mediated passive systemic anaphylaxis (182) (**Table 3**). In recent clinical trials, lirentelimab showed positive effects in the treatment of patients with asthma and eosinophilic gastroenteritis (135, 231), and further clinical applications are currently under investigation, albeit not for food allergy (**Table 2**).

Within the CD300 receptor family, only CD300a and CD300f show ITIM/ITIM-like domains, expressed on MCs. In humans, CD300 receptor ligands include phosphatidylserine (CD300a), ceramide, sphingomyelin (CD300f), released by apoptotic, tumor or infected cells (214). In addition to the disruption of IgE-mediated activation (179), CD300a engagement also impairs MC proliferation and survival by inhibiting stem cell factor (SCF) signaling (232), whereas co-engagement of CD300f with IL-4Rα promotes IL-4 mediated activation of MCs (233). Fusion proteins targeting CD300a and IgE on MCs in a passive cutaneous anaphylaxis mouse model, showed a successful reduction in MC activation (179) (**Table 3**).

## Allergen-Dependent Approaches

### Modulation of Mast Cell Reactivity Using Allergen-Specific Immunotherapy

Allergen-specific immunotherapy (AIT) is the only disease-modifying intervention currently available to treat some allergic conditions, like insect venom allergy, allergic rhinitis and asthma due to respiratory allergy to pollens and house dust mites (234–237).

AIT consists in the repetitive exposure to escalating doses of native allergen extracts, which might induce generalized MC and basophil activation. The risk of eliciting an anaphylactic episode is mitigated by starting with very low allergen doses, by being performed only by trained professionals and in safe conditions under careful monitoring of potential early signs of systemic reaction (236, 238). The timing of dose increase depends on the protocol, ranging from weeks in conventional AIT to days/hours in rush/ultra-rush protocols (234, 238).

The concerted activity of cells from both innate and acquired immunity contributes to the efficacy of AIT (34–36), ultimately eliciting antigen-selective inhibition of MC and basophil activation and long-lasting suppression of IgE-mediated responses at large. In fact, AIT induces a pro-tolerogenic state, promoting allergen-specific IgG/IgG4 production opposed to sIgE by B cells (15). IgG and IgG4 not only selectively compete with IgE in allergen binding, but also the engagement of the FcγRIIb receptor by allergen-IgG complexes cross-linking with surface IgE-FcϵRI actively inhibits MC activation (218, 239–242). IgG-mediated inhibition also prevents further amplification of IgE production, by reducing Th2 cytokine release from activated MCs and basophils (242).

AIT also promotes the development of Tregs, which suppress MC activities, not only by secreting the anti-inflammatory cytokine IL-10, but also inducing MC cell anergy *via* OX40L receptor engagement (15, 27, 243). OX40-OX40L binding on MCs activates downstream signalling by C-terminal Src kinases, suppressing Fyn kinase activity and impairing microtubule rearrangement and degranulation (243) (**Figure 1E**).

Although effective, these events require time to induce a protective response, while exposure to incremental doses of allergen rapidly desensitizes MCs. However, the mechanism explaining such effect remains unclear. A study suggests that rapid incremental IgE receptor occupancy induces the depletion of cell surface IgE by internalization of IgE–FcϵRI complexes (244). Others find in desensitized anergic MCs an impaired internalization of allergen–IgE–FcϵRI molecules (245), and aberrant rearrangements of cytoskeleton actin fibers that inhibit FcϵRI-mediated calcium flux and intracellular vesicles trafficking (246).

Rapidly desensitized MCs, in turn, produce IL-2 that contribute to Treg survival and recruitment in the periphery, hence indirectly contributing to peripheral tolerance, as demonstrated in mice (247).

Both tolerance induction and MC desensitization are widely exploited to achieve long-term modulation and quick onset protection of allergic reactions with rush/ultra-rush protocols, respectively (248).

### Allergen-Specific Immunotherapy in Food Allergy

For both treatment and prevention of severe reactions upon accidental exposure to food allergens, increasing the maximum tolerated dose of allergen is necessary and can be achieved with AIT (249).

AIT in food allergy is performed using either native allergens (e.g. whole food, allergen extracts) administered *via* the oral, sublingual or epicutaneous routes, or baked allergens (alone or mixed with other ingredients creating a food matrix) *via* the oral route (250).

Recently, the first peanut allergen powder formulation (AR101) was approved for peanut AIT by the U.S. Food and Drug Administration and European Medicines Agency (251, 252), and numerous other trials using either whole peanut or peanut extracts promoted tolerance to varying doses of crude peanut in 60–80% of treated subjects (72, 81, 83, 85, 90, 92, 94) (**Table 1**). However, the safety of AIT protocols in food allergy is still a matter of debate, since the risk of a severe allergic reaction during AIT cannot be completely abated (253). In fact, a 1–21% frequency of systemic adverse reactions and increased occurrence with higher peanut end goal doses were observed in peanut AIT trials (254). Furthermore, while long-term treatment is effective in preventing severe allergic reactions in AIT responders (79, 92), a fraction of subjects might still experience anaphylaxis with previously tolerated allergen doses when aggravating co-factors are present (i.e. physical exercise, use of non-steroidal anti-inflammatory drugs, infections, etc.) or due to poor AIT adherence (79, 253).

### Combination Treatments With Biologics

To increase AIT safety in food allergy, newer therapeutic strategies involve the combination of AIT with biologics. Evidence suggests that omalizumab administered during AIT reduces the risk of severe reactions and facilitates AIT (97, 99, 101) (**Tables 1** and **2**). In fact, while AIT caused an increase in the levels of inhibitory allergen-specific IgG4, in the threshold for MC responsiveness and a reduction of Th2 cytokine production (83, 84, 92, 239), omalizumab decreased the likelihood of basophil degranulation, especially relevant during dose escalation (101). This omalizumab-induced protection is most likely dependent on basophil IgE–FcϵRI disengagement, as suggested by empirical evidence (159) and omalizumab pharmacokinetics.

However, studies on long-term use of omalizumab in cow’s milk AIT proved long-term omalizumab add-on treatment not being cost-effective, albeit the higher safety profile (255) (**Table 2**). Further trials testing the utility of omalizumab adjunct to food AIT, or other biologics like dupilumab with AR101 (NCT03682770) are currently ongoing (**Tables 1** and **2**).

### Alternative Food Immunotherapy Approaches

Allergen-dependent strategies alternative to AIT are currently being tested. Among these, the use of hypoallergenic molecules, lacking key anaphylactogenic conformational epitopes, promises to obtain safer alternatives to AIT using native allergen extracts, as observed in fish and peanut allergy studies conducted in humans and mice, albeit still in early development (256, 257).

Other therapeutic approaches involve antibodies targeting major allergenic molecules, like a recently developed monoclonal anti-Ara h 2, preventing both local and systemic allergic reactions, as tested in a mouse model of peanut allergy (177) (**Table 3**). The advantage of monoclonal treatment is not only given by their competition with IgE molecules in allergen binding, but also by sharing with endogenous allergen-specific IgG antibodies the same mechanisms, regardless of patients’ capacity to mount an effective anti-allergic immune response as in conventional allergen immunotherapy. However, subjects sensitized to multiple allergen epitopes might only partially benefit from such treatment, unless multiple monoclonal antibodies against different epitopes are used in combination.

The complexing of allergenic epitopes with molecules actively promoting a tolerogenic state (i.e. production of IL-10, induction of IgG4, generation of Tregs), such as Toll-like receptor ligands (i.e. CpG, LPS, R848), viral-attenuated molecules, Siglec-engaging tolerance-inducing antigenic liposomes (STALs) and nanoformulations, is used as adjuvant immunotherapy to elicit allergen-specific tolerance (258).

An alternative approach under study is the use of plasmid DNA-based vaccines. Such vaccines induce the production of specific exogenous target proteins *via* allergen-coding DNA particles, exploiting the natural immune pathways leading to the production of IgG to promote long-lasting tolerance (259). In addition, peptide vaccines aimed at eliciting IgG antibody production targeted against highly allergenic epitopes are also currently under scrutiny (260).

Several recent studies on nanoformulations and adjuvant immunotherapy candidates for cow’s milk and peanut allergy have been conducted, showing promising results in mouse allergy models (183, 184, 261, 262) (**Table 3**). In humans, few ongoing clinical trials on DNA-based vaccines (ASP0892, NCT03755713; ASP0892, NCT02851277) and modified allergen proteins (HAL-MPE1 subcutaneous AIT, NCT02991885) are currently in Phase I, while a previous attempt with attenuated *E. Coli* Ara h 1-2-3 recombinant vaccine candidate failed to promote tolerance, inducing severe adverse reactions in 20% of participants (96) (**Table 1**).

## Conclusions

Albeit complex, the allergic immune response relies on MC functionality, making these cells important targets for therapeutic intervention. Given the plethora of current and future treatments, some considerations on most promising choices and benefit/risk assessment are warranted.

Anti-IgE treatment is a valuable option for the control of food allergy symptoms and especially beneficial when adjunct to AIT. The lack of specificity and long term use of anti-IgE treatment was historically considered a concern, due to the loss of the protective IgE housekeeping functions. However, after 20+ years of omalizumab use, no increased risks for parasitic or neoplastic events could be observed (185, 263). Apart from a negligible risk of anaphylaxis upon the first administrations (264), omalizumab has been successfully used for long-term treatment and during pregnancy with an excellent safety profile (265). However, limited data is currently available on its safety in children less than 6 years of age, hence narrowing its therapeutic range.

AIT and allergen-specific vaccines are currently the only allergen-dependent interventions showing a curative potential in food allergy, however the risks associated to the exposure to allergenic molecules for treatment purposes should be minimized as much as possible, with safer protocols and drug formulations.

While allergen-dependent therapeutic strategies require the full functionality of the immune system to work, showing great variability in treatment response between individuals, sIgE inhibition could hamper allergen-specific activation regardless of the quality of patients’ immune response, but likely without comparable long-term disease-modifying effect as AIT.

The engagement of inhibitory receptors, abundantly expressed and not unique to MCs, are not only effectively inhibiting MC functions, but their activities can be directed against specific epitopes by formulating bispecific allergen-inhibitory ligand molecules [e.g. CD33L-coated liposomal nanoparticles (180), Ara h 2-FcγRIIb fusion proteins (175)]. This envisages a targeted allergen-specific inhibitory approach, while preserving pathways for IgE-mediated housekeeping functions, albeit still in early development.

Given the wide distribution of cytokine receptors and their pleiotropic effects exerted on many different cell types, therapeutic strategies blocking IL-4Rα, or cytokines important for the initiator phase of immune responses, like IL-33 and TSLP, pose some concerns. The suppression of protective immunity, the generation of paradoxical responses as, for instance, the conjunctivitis induced by dupilumab treatment in atopic dermatitis (266), or the little known effects of long-term exposure are safety issues that need further clarification.

Conversely, the broad, simultaneous and unspecific inhibition of multiple effector cells involved in allergic responses by anti-cytokine or by anti-Siglec monoclonal antibodies is potentially beneficial in the modulation of complex inflammatory diseases, as observed in asthma, atopic dermatitis, chronic rhinosinusitis with nasal polyps, eosinophilic gastroenteritis and other Th2-mediated conditions, including food allergy (**Tables 1** and **2**). Therefore, both anti-cytokine and anti-Siglec monoclonal antibodies are among the most encouraging disease-modifying allergen-independent therapies available in the near future for the treatment of severe allergic conditions, warranting further consideration especially in the field of food allergy.

Despite that there is still a strong need for clinical trials to assess the efficacy and safety of both allergen-independent and -dependent therapeutic approaches, the knowledge on the immunological mechanisms behind MC activation are the ultimate key for a successful allergy therapeutic intervention.

## Author Contributions 

CT and SB-P reviewed literature and wrote the article. All authors contributed to the article and approved the submitted version.

## Funding

CT is funded by the UK Research and Innovation (UKRI) Medical Research Council Doctoral Training Partnership (MRC-DTP) studentship. SB-P received funding from MRC (MR/S036954/1).

## Conflict of Interest

The authors declare that the research was conducted in the absence of any commercial or financial relationships that could be construed as a potential conflict of interest.

## Publisher’s Note

All claims expressed in this article are solely those of the authors and do not necessarily represent those of their affiliated organizations, or those of the publisher, the editors and the reviewers. Any product that may be evaluated in this article, or claim that may be made by its manufacturer, is not guaranteed or endorsed by the publisher.

## Abbreviations

AIT, allergen immunotherapy; Akt, protein kinase B; BTK, Bruton Tyrosine Kinase; ERK, extracellular signal-regulated kinase; FcϵRI, high-affinity IgE receptor; FcϵRII, low affinity IgE receptor; FcγRII, low affinity IgG receptor; IgE, immunoglobulin E; IgG, immunoglobulin G; IL, interleukin; IL-4Rα, interleukin 4 receptor alpha chain; ITAM, immunoreceptor tyrosine-based activation motif; ITIM, immunoreceptor tyrosine-based inhibition motif; Kd, dissociation constant; LARI, low affinity allergic response inhibitors; MCs, mast cells; NF-k, B, Nuclear Factor kappa-light-chain-enhancer of activated B cells; PI3K, phosphatidylinositol 3-OH kinase; SCF, stem cell factor; Siglec, Sialic acid-binding immunoglobulin-type lectins; sIgE, allergen-specific immunoglobulin E; STAT6, signal transducer and activator of transcription 6; Syk, Spleen Tyrosine Kinase; TSLP, Thymic stromal lymphopoietin; Tregs, T regulatory cells.
